# Synthesis and Spectroscopic Analysis of Novel 1*H*-Benzo[*d*]imidazoles Phenyl Sulfonylpiperazines 

**DOI:** 10.3390/ph5050460

**Published:** 2012-05-03

**Authors:** Amjad M. Qandil

**Affiliations:** 1 Pharmaceutical Sciences Department, College of Pharmacy, King Saud bin Abdulaziz University for Health Sciences, Riyadh, 11426, Saudi Arabia; Email: Qandila@ksau-hs.edu.sa; Tel.: +966-1-252-00-88 (ext. 51091); Mobile: +966-5-68-93-81-82; 2 Department of Medicinal Chemistry and Pharmacognosy, Faculty of Pharmacy, Jordan University of Science and Technology, Irbid 22110, Jordan; Email: drqandil@just.edu.jo

**Keywords:** phenyl sulfonylpiperazine, benzimidazole, mass spectrometry, NMR

## Abstract

A group of benzimidazole analogs of sildenafil, 3-benzimidazolyl-4-methoxy-phenylsulfonylpiperazines **2–4 **and 3-benzimidazolyl-4-methoxy-*N,N*-dimethyl- benzenesulfonamide (**5**), were efficiently synthesized. Compounds **2–5** were characterized by NMR and MS and contrary to the reported mass spectra of sildenafil, the spectra of the piperazine-containing compounds **2–4** showed a novel fragmentation pattern leading to an *m/z* = 316. A mechanism for the formation of this fragment was proposed.

## 1. Introduction

**Figure 1:**
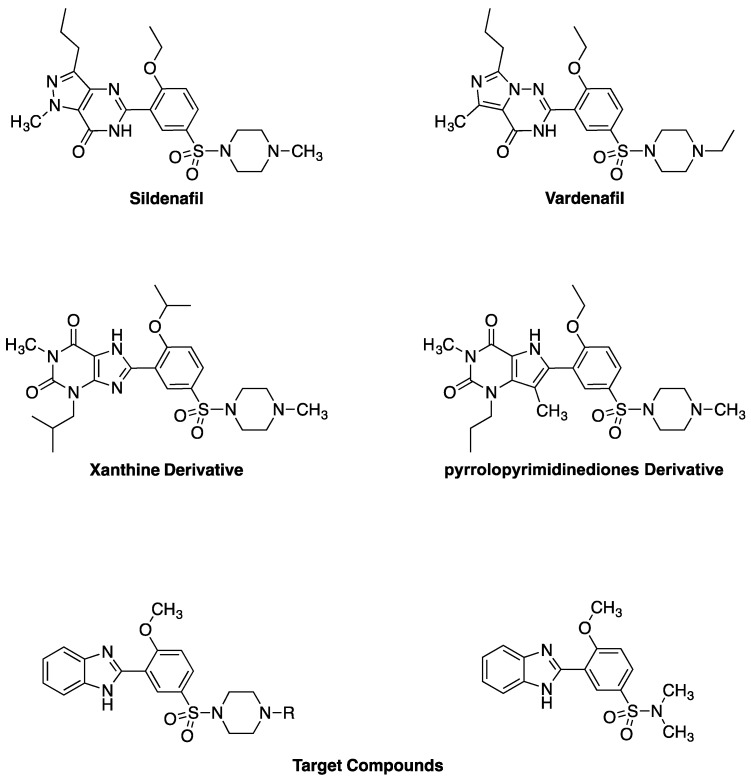
The chemical structures of sildenafil, vardenafil, xanthine derivative, pyrrolopyrimidinedione derivative and the target compounds.

Substituted-phenylsulfonylpiperazines such as sildenafil and vardenafil (Figure 1), are being marketed for the treatment of erectile dysfunction because of their phosphodiesterase inhibitor-5 inhibitory activity [1,2,3,4,5]. Ordonafil, a cyclic ether analog of sildenafil, has also been shown to prevent myocardial hypertrophy [6]. Other substituted-phenylsulfonylpiperazines have adenosine A_2B_ receptor antagonistic activity [7], anti-inflammatory activity [8] and IKK2 inhibitory activity [9]. 

In general, the synthesis of sildenafil and its closely related analogs requires a long multistep synthetic pathway, which is due, in part, to the presence of the aromatic heterocyclic ring [10] and only became less tedious after 4-amino-4,5-dihydro-1-methyl-3-propyl-1*H*-pyrazole-5-carboxamide became commercially available [1]. Other analogs containing similar and sometimes more complicated heterocyclic aromatic systems have been reported, namely, imidazolotriazinones [11], xanthines [12], pyrrolopyrimidinediones [13], imidazoquinazoline [14], pyridopurinone [15] and triazolopurinones [13]. The xanthine and pyrrolopyrimidinedione derivatives (Figure 1), in which the phenyl-sulfonylpiperazine is connected to the five membered ring of the aromatic system rather than the six member ring as in sildenafil, have shown nanomolar inhibitory activity against PDE-5 [12,13]. Herein, simpler benzimidazole analogs of sildenafil that are related to xanthines and pyrrolopyrimidinediones are reported. The target compounds have the general formulas 3-benzimidazolyl-3-methoxyphenyl-sulfoylpiperazines and 3-benzimidazolyl-4-methoxy-*N,N*-dimethylbenzenesulfonamide. The synthesis proceeded using readily available inexpensive starting materials and reagents and the synthesized compounds were characterized by ^1^H-NMR, ^13^C-NMR and Mass Spectrometry.

## 2. Results and Discussion

**Scheme 1:**
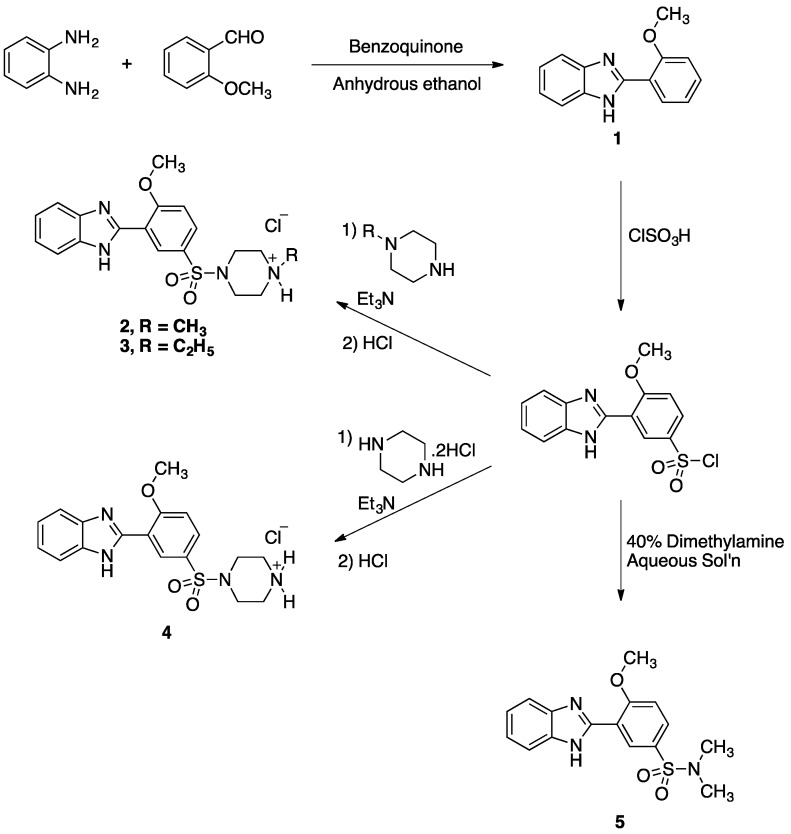
The synthetic pathway for the preparation of compounds **2–5**.

We were interested in an efficient, fast and inexpensive synthesis of the target compounds. The primary concern was to find and then execute a simple one-pot procedure for the preparation of the tricyclic benzimidazole backbone starting from *o*-anisaldehyde. Many procedures which can attain this goal have been reported in recent literature. For example, the treatment of the appropriate aldehyde with an *ortho-*diaminobenzene in the presence of an oxidizing agent can afford the corresponding benzimidazole [16,17,18,19,20]. Also, the treatment of *o*-nitroanilines with aldehydes in the presence of Na_2_S_2_O_4_ can also afford benzimidazoles [21]. Another procedure called for the treatment of 2-halo-anilines with aldehydes in the presence of copper (I) chloride and sodium azide [22]. For this work, an addition-oxidation of phenylenediamine to *o*-anisaldehyde in the presence of 1,4-benzoquinone, as an oxidizing agent, was employed to afforded the desired benzimidazole **(1)** in good yields [23]. It was critical to add all the starting materials together and wait for 30 min before adding the solvent, ethanol. The benzimidazole **1** was then chlorosulfonated selectively at the *para*-position of the anisyl moiety and the resultant sulfonyl chloride was extensively washed with water and used in the next step without further purification. To obtain the final compounds, the sulfonyl chloride, dissolved in dichloromethane, was coupled to 1-methylpiperazine, *N*-ethylpiperazine and piperazine dihydrochloride in the presence of excess amounts of triethylamine [24,25]. The resultant amines were then converted to the hydrochloride salts and purified by crystallization from methanol/ethyl acetate to afford compounds **2, 3 **and **4** in good yields. To obtain compound **5**, the sulfonyl chloride was treated with an aqueous solution of dimethylamine and the product was crystallized form ethyl acetate in good yields. The synthetic pathway for the preparation of target compounds **2–5** is illustrated in Scheme 1.

**Scheme 2:**
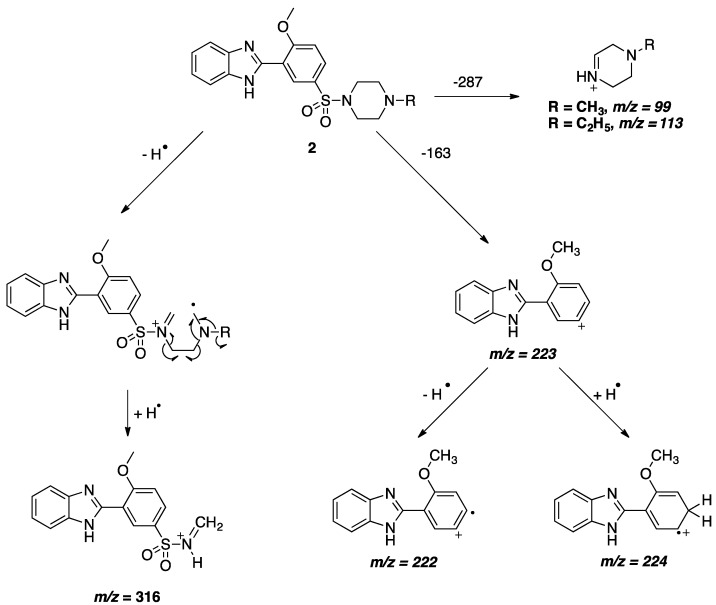
The fragmentation scheme of compounds **2–4**.

Compounds **1–5** were characterized by ^1^H-NMR, ^13^C-NMR and Mass Spectrometry. While the NMR and mass spectra have confirmed the identity of these compounds, there was a peak in the mass spectra of compounds **2–4** that caught our attention. The *m/z* = 316 was present with medium to high intensities, 52-97%, in the mass spectra of the piperazine ring-containing compounds only, although, it was expected that compound **5** would be the one to form this *m/z* by simple cleavage of one of the methyl groups on the dimethylsulfonamide moiety, but such *m/z* was not present in its spectrum. This peak could not be explained by simple fragmentation and hence a possible scheme for the formation of this fragment is proposed, as seen in Scheme 2, in accordance with the work of Shetty *et al.* on the fragmentation of piperazine-containing phenothiazine antispcyhotic agents [26]. The unique mass spectra of the herein reported compounds **2–4** can be considered diagnostic for their identity. In addition, it is worth mentioning that there are reports of detailed MS analyses of sildenafil and other chemically similar compounds [27,28,29] but none of these reports suggested this pattern of fragmentation, which led to the belief that it is novel for these phenylsulfonylpiperazines. Other fragments that were present and their molecular formulae are also shown in Scheme 2.

## 3. Experimental

### 3.1. Materials

Bulk solvents were purchased through local vendors. Reagent grade and fine chemicals were obtained from, Aldrich Chemical Company, Germany (www.sigmaaldrich.com), ACROS Chemicals, Belgium (www.acros.com) and Scharlau Chemicals, Spain (www.scharlau.com). Melting points were determined using Stuart Scientific melting point apparatus (Stuart Scientific, Stone, Staffordshire, UK) and were uncorrected. NMR spectra were acquired using a Bruker Advance Ultrashield 400 MHz instrument, (Bruker, Fallanden Switzerland) and chemical shifts (δ) were reported in ppm relative to automatic calibration to the residual proton peak of the solvent used namely DMSO-*d*_6_. TLC analysis was performed on Merck aluminum TLC plates, Silica 60, F_254_, (Merck, Darmstadt, Germany). The electron impact (EI) mass spectra were obtained using a Shimadzu QP5050A GC-MS spectrometer (Japan) by direct injection.

### 3.2. Synthesis

#### 3.2.1. Synthesis of 2-(2-methoxyphenyl)-1H-benzo[d]imidazole (**1**)

To phenylenediamine (15.0 g, 138.71 mmol), *o-*anisaldehyde (18.9 g, 138.88 mmol) and 1,4-benzo-quinone (15.0 g, 138.77 mmol) were added. After 30 min, the mixture was dissolved in anhydrous ethanol (300 mL) and the reaction mixture was heated at reflux temperature for three hours. The solvent was then evaporated and the residue was suspended in 1N sodium hydroxide (300 mL) and then filtered and dissolved in ethyl acetate and the solution was dried over magnesium sulfate. The solvent was then evaporated and the residue was crystallized from ethyl acetate to afford 2-(2-methoxyphenyl)-1*H*-benzo[*d*]imidazole **(1)** (16.70 g, 74.47 %) as an off-white solid. MS: *m/z*: 224 (100%), 223 (63%), 194 (61%), 119 (54%). ^1^H-NMR (400 MHz, DMSO-*d*_6_): δ 4.00 (s, 3H, OCH_3_), 7.10 (t, 1H, *J* = 7.5 Hz); 7.19 (m, 3H); 7.46 (td, 1H, , *J* = 7.8, 1.8 Hz); 7.62 (m, 2H); 8.30 (dd, 1H, *J* = 7.8, 1.8 Hz); 8.68 (s, 1H) and 12.15 (s, 1H, NH). ^13^C-NMR (100 MHz, DMSO-*d*_6_): δ 56.19, 112.40, 112.52, 118.51, 118.86, 121.33, 122.01, 125.45, 130.19, 131.73, 135.15, 143.12, 149.40, 150.19 and 157.20.

#### 3.2.2. General Procedure for the Synthesis of compounds **2** and **3**

2-(2-Methoxyphenyl)-1*H*-benzo[*d*]imidazole (**1**) (3.00 g, 13.38 mmol) was suspended in ice-cooled chlorosulfonic acid (10 mL) and the mixture was stirred at that temperature for one hour. The reaction mixture was then added to crushed ice and filtered and was extensively washed with water. The solid residue was suspended in dichloromethane (50 mL) and to it triethylamine (4.14 g, 5.7 mL) and 1-methylpiperazine or 1-ethylpiperazine (1 eq) were added sequentially and the mixture was stirred for 3 hrs. Then, the reaction mixture was washed with water and then dried over magnesium sulfate and the solvent was evaporated. The residue was then converted to the hydrochloric acid salt and crystallized from methanol/ethyl acetate to afford compounds **2 **and **3**.

*2-(2-Methoxy-5-((4-methylpiperazin-1-yl)sulfonyl)phenyl)-1H-benzo[d]imidazole hydro-chloride* (**2**): 1-Methylpiperazine (1.34 g, 1.50 mL, 13.38 mmol) was used to prepare **2** (4.12 g, 72.80%) as white crystals. MS: *m/z*: 316 (97%), 99 (100%), 56 (65%). ^1^H-NMR (400 MHz, DMSO-*d*_6_): δ 2.72 (s, 3H, NCH_3_); 2.87 (t, 2H, *J* = 12.3 Hz); 3.17 (t, 2H, *J* = 12.3 Hz); 3.42 (d, 2H, *J* = 12.5 Hz); 3.90 (d, 2H, *J* = 12.5 Hz); 4.17 (s, 3H, OCH_3_); 7.55 (m, 2H); 7.64 (m, 3H); 7.46 (d, 1H, *J* = 9.0 Hz); 7.9 (m, 2H); 8.07 (dd, 1H, *J* = 9.0, 2.3 Hz); 8.73 (d, 1H, *J* = 2.3 Hz) and 11.43 (s, 1H, NH). ^13^C-NMR (100 MHz, DMSO-*d*_6_): δ 42.27, 43.50, 51.80, 57.84, 113.31. 114.53, 114.84, 126.32, 127.95, 130.86, 132.38, 134.19, 144.79, 161.33.

*2-(5-((4-Ethylpiperazin-1-yl)sulfonyl)-2-methoxyphenyl)-1H-benzo[d]imidazole hydro-chloride* (**3**): 1-Ethylpiperazine (1.53 g, 1.70 mL, 13.38 mmol) was used to prepare **3** (3.94 g, 67.40%) as white crystals. MS: *m/z*: 316 (87%), 113 (100%), 56 (73%). ^1^H-NMR (400 MHz, DMSO-*d*_6_): δ 1.19 (s, 3H, CH_3_); 2.92 (s, 3H, NCH_3_); 3.10 (m, 4H); 3.49 (d, 2H, *J* = 12.2 Hz); 3.89 (d, 2H, *J* = 12.6 Hz); 4.17 (s, 3H, OCH_3_); 7.55 (m, 2H); 7.64 (dd, 2H, *J* = 9.1, 1.4 Hz); 7.91 (m, 2H); 8.07 (d, 1H, *J* = 9.1 Hz); 8.73 (s, 1H) and 11.31 (s, 1H, NH). ^13^C-NMR (100 MHz, DMSO-*d*_6_): δ 9.09, 43.54, 49.77, 50.92, 57.86, 113.19. 114.53, 114.81, 126.37, 127.89, 130.83, 132.26, 134.27, 144.69, 161.35.

#### 3.2.3. 2-(2-methoxy-5-((4-piperazin-1-yl)sulfonyl)phenyl)-1*H*-benzo[*d*]imidazole hydrochloride (**4**)

2-(2-Methoxyphenyl)-1*H*-benzo[*d*]imidazole **(1)** (3.00 g, 13.38 mmol) was suspended in ice-cooled chlorosulfonic acid (10 mL) and the mixture was stirred at that temperature for one hour. The reaction mixture was then added to crushed ice and filtered. The solid residue was suspended in dichloromethane (50 mL) and to it triethylamine (15.53 g, 20.00 mL) and piperazine dihydrochloride (15.91 g, 100.03 mmol) were added sequentially and the mixture was stirred for 3 hrs. Then, the reaction mixture was washed with water and then dried over magnesium sulfate and the solvent was evaporated. The residue was then converted to the hydrochloric acid salt and crystallized from methanol/ethyl acetate to afford compound **4** (4.76 g, 84.20 %) as white crystals. MS: *m/z*: 316 (52%), 224 (100%), 56 (96%). ^1^H-NMR (400 MHz, DMSO-*d*_6_): δ 3.16 (bs, 4H); 3.29 (bs, 4H); 4.17 (s, 3H, OCH_3_); 7.55 (m, 2H); 7.64 (d, 2H, *J* = 9.0 Hz); 7.91 (m, 2H); 8.06 (dd, 1H, *J* = 9.0, 2.3 Hz); 8.68 (d, 1H, *J* = 2.3 Hz) and 9.97 (s, 1H, NH). ^13^C-NMR (100 MHz, DMSO-*d*_6_): δ 42.45, 43.31, 57.88, 113.19. 114.52, 114.82, 126.39, 127.84, 130.84, 132.27, 134.32, 144.70, 161.2.

#### 3.2.4. 3-(1*H*-Benzo[*d*]imidazol-2-yl)-4-methoxy-*N*,*N*-dimethylbenzenesulfonamide (**5**)

2-(2-Methoxyphenyl)-1*H*-benzo[*d*]imidazole (**1**, 3.00 g, 13.38 mmol) was suspended in ice-cooled chlorosulfonic (10 mL) and the mixture was stirred at that temperature for one hour. The reaction mixture was then added to crushed ice and filtered. The solid residue was suspended in 40% aqueous dimethylamine (50 mL) and stirred for 1 hr. Then, the reaction mixture was filtered and allowed to dry. The dry residue was crystallized from ethyl acetate to afford compound **5** (3.49 g, 78.60 %) as off-white crystals. MS: *m/z*: 331 (80%), 224 (75%), 222 (100%), 56 (96%). ^1^H-NMR (400 MHz, DMSO-*d*_6_): δ 2.67 (s, 6H, N(CH_3_)_2_); 3.58 (s, 3H, OCH_3_); 7.56 (m, 2H); 7.62 (d, 2H, *J* = 8.9 Hz); 7.91 (m, 2H); 8.04 (dd, 1H, *J* = 8.9, 2.3 Hz); 8.59 (d, 1H, *J* = 2.3 Hz). ^13^C-NMR (100 MHz, DMSO-*d*_6_): δ 38.35, 57.74, 112.91. 114.25, 114.79, 126.39, 127.93, 130.51, 132.19, 134.34, 144.97, 160.98.

## 4. Conclusions

Four benzimidazole analogs of sildenafil have been successfully synthesized using a straightforward, short and cost effective pathway. The reaction times were 3 h or less and yields were 67.40-84.20%. The identity of the synthesized compounds was verified using NMR and MS analyses. The MS analysis of the piperazine containing compounds showed a novel fragmentation pattern for phenyl sulfonylpiperzines leading to the formation of a diagnostic peak with *m/z* = 316. A mechanism for the formation of the characteristic *m/z* (316) was proposed and the molecular formulas of the most prominent peaks in the mass spectra of the synthesized compounds, 99, 113, 222, 223 and 224, have been proposed.

## Correction

Correction A correction was published on 24 September 2012: http://www.mdpi.com/1424-8247/5/9/1044 (PDF, 110 KB)
