# Qandil, A.M., Synthesis and Spectroscopic Analysis of Novel 1*H*-Benzo[d]imidazolylphenylsulfonylpiperazines. *Pharmaceuticals* 2012, *5*, 460-468

**DOI:** 10.3390/ph5091044

**Published:** 2012-09-24

**Authors:** Amjad M. Qandil

**Affiliations:** 1 Pharmaceutical Sciences Department, College of Pharmacy, King Saud bin Abdulaziz University for Health Sciences, Riyadh, 11426, Saudi Arabia; Email: Qandila@ksau-hs.edu.sa; Tel.: +966-1-252-00-88 (ext. 51091); Mobile: +966-5-68-93-81-82; 2 Department of Medicinal Chemistry and Pharmacognosy, Faculty of Pharmacy, Jordan University of Science and Technology, Irbid 22110, Jordan; Email: drqandil@just.edu.jo

We have found the following errors in the title of this article which was recently published in Pharmaceuticals [1]:

1. The correct title should be: **Synthesis and Spectroscopic Analysis of Novel 1*H*-Benzo[d]imidazolylphenylsulfonylpiperazines**.
2. The phrase “phenyl sulfonylpiperazines” should be replaced by “phenylsulfonylpiperazines” whenever mentioned in the article, namely in the Keywords, page 460, and in the penultimate line of page 465.
