# Supplementary material for: Synthesis and Spectroscopic Analysis of Novel 1H-Benzo[d]imidazoles Phenyl Sulfonylpiperazines
Source: Pharmaceuticals (Basel). 2012 May 3;5(5):460–8. doi: 10.3390/ph5050460 (PMC3763652; doi:10.3390/ph5050460)
Supplement: Correction — A correction was published on 24 September 2012: http://www.mdpi.com/1424-8247/5/9/1044 (PDF, 110 KB) [file pharmaceuticals-05-00460-s001.pdf]

Pharmaceuticals **2012**, *5*, 1044; doi:10.3390/ph5091044

OPEN ACCESS

pharmaceuticals

ISSN 1424-8247

www.mdpi.com/journal/pharmaceuticals

Correction

Qandil, A.M., Synthesis and Spectroscopic Analysis of Novel 1*H*-Benzo[d]imidazolylphenylsulfonylpiperazines. *Pharmaceuticals* 2012, *5*, 460-468

Amjad M. Qandil [1,2]

[1] Pharmaceutical Sciences Department, College of Pharmacy, King Saud bin Abdulaziz University for Health Sciences, Riyadh, 11426, Saudi Arabia; E-Mail: Qandila@ksau-hs.edu.sa; Tel.: +966-1-252-00-88 (ext. 51091); Mobile: +966-5-68-93-81-82

[2] Department of Medicinal Chemistry and Pharmacognosy, Faculty of Pharmacy, Jordan University of Science and Technology, Irbid 22110, Jordan; E-Mail: drqandil@just.edu.jo

Received: 18 September 2012 / Published: 24 September 2012

We have found the following errors in the title of this article which was recently published in Pharmaceuticals [1]:

1. The correct title should be: **Synthesis and Spectroscopic Analysis of Novel 1*H*-Benzo[d]imidazolylphenylsulfonylpiperazines**.
2. The phrase "phenyl sulfonylpiperazines" should be replaced by "phenylsulfonylpiperazines" whenever mentioned in the article, namely in the Keywords, page 460, and in the penultimate line of page 465.

Reference

1. Qandil, A.M. Synthesis and Spectroscopic Analysis of Novel 1H-Benzo[d]imidazoles Phenyl Sulfonylpiperazines. *Pharmaceuticals* **2012**, *5*, 460-468

© 2012 by the authors; licensee MDPI, Basel, Switzerland. This article is an open access article distributed under the terms and conditions of the Creative Commons Attribution license (http://creativecommons.org/licenses/by/3.0/).
